# Compliance with guidelines is related to better local recurrence-free survival in ductal carcinoma *in situ*

**DOI:** 10.1038/sj.bjc.6602815

**Published:** 2005-10-18

**Authors:** M A J de Roos, G H de Bock, P C Baas, L de Munck, T Wiggers, J de Vries

**Affiliations:** 1 Department of Surgical Oncology, University Medical Center Groningen, University of Groningen, Hanzeplein 1, PO Box 30001, 9700 RB Groningen, The Netherlands; 2 Department of Epidemiology and Bioinformatics, University Medical Center Groningen, University of Groningen, Groningen, The Netherlands; 3 Department of Surgery, Martini Hospital, van Swietenlaan 4, PO Box 30033, 9700 RM Groningen, The Netherlands; 4 Comprehensive Cancer Center North Netherlands, Waterloolaan 1/13, PO Box 330, 9700 AH Groningen, The Netherlands

**Keywords:** breast neoplasm, ductal carcinoma *in situ*, clinical guidelines, breast-conserving surgery, mastectomy, local neoplasm recurrence

## Abstract

The aim was to study the effect of compliance with guidelines on local recurrence (LR)-free survival in patients treated for ductal carcinoma *in situ* (DCIS). From January 1992 to December 2003, 251 consecutive patients had been treated for DCIS in two hospitals in the North Netherlands. Every case in this two-hospital sample was reviewed in retrospect for its clinical and pathological parameters. It was determined whether treatment had been carried out according to clinical guidelines, and outcomes in follow-up were assessed. In addition, all patients treated for DCIS in this region (*n*=1389) were studied regarding clinical parameters, in order to determine whether the two-hospital sample was representative of the entire region. In the two-hospital sample, 31.4% (*n*=79) of the patients had not been treated according to the guidelines. Positive margins were associated with LR (hazard ratio (HR)=4.790, 95% confidence interval (CI) 1.696–13.531). Breast-conserving surgery and deviation from the guidelines were independent predictors of LR (HR=7.842, 95% CI 2.126–28.926; HR=2.778, 95% CI 0.982–6.781, respectively). Although the guidelines changed over time, time was not a significant factor in predicting LRs (HR=1.254, 95% CI 0.272–5.776 for time period 1992–1995 and HR=1.976, 95% CI 0.526–7.421 for time period 1996–1999). Clinical guidelines for the treatment of patients with DCIS have been developed and updated from existing literature and best evidence. Compliance with the guidelines was an independent predictor of disease-free survival. These findings support the application of guidelines in the treatment of DCIS.

The main goal in the treatment of DCIS is the prevention of local recurrences (LR). Standards regarding the optimal management of patients with DCIS have been developed (Schwartz *et al*, 2000; Morrow *et al*, 2002; Mokbel 2003; Sakorafas and Farley 2003; Burstein *et al*, 2004). Mastectomy could be performed in cases with multiple areas of DCIS and in cases with large areas of DCIS, of a size that the lesion cannot undergo an oncologically and cosmetically acceptable excision (⩾3 cm (Sakorafas and Farley, 2003); ⩾4 cm (Mokbel, 2003)). Mastectomy could also be considered in cases with positive margins after reasonable surgical attempts of complete local excision or if it were the patient's preference and it may be indicated when breast irradiation is contraindicated (e.g. in patients who are pregnant or have collagen vascular disease). Breast-conserving surgery (BCS) could be considered where DCIS is localised and if the area ⩽4 cm. Removal of DCIS should be complete and margins should be at least 1 mm free of DCIS (Burstein *et al*, 2004). The addition of radiotherapy following local excision benefits all groups of patients with DCIS and is generally advised. Neither axillary-node dissection nor sentinel node biopsy (SNB) is routinely indicated. SNB could be considered in women undergoing mastectomy or in women at higher risk of occult invasive disease (patients with clinically palpable masses or areas of DCIS >4 cm). Adjuvant endocrine therapy could be considered after BCS with radiotherapy to reduce the risk of ipsilateral recurrence and contralateral disease, particularly in the case of oestrogen receptor-positive DCIS (Morrow *et al*, 2002; Burstein *et al*, 2004).

The purpose of such retrospective study is to evaluate the effect of compliance with existing clinical standards on LR-free survival in patients treated for DCIS. To estimate the effect of compliance with clinical standards in this two-hospital study, the clinical and pathological parameters for every consecutive case of DCIS, in a given period, were examined retrospectively. It was determined whether treatment had been carried out according to the standards existing at the time and the outcomes in follow-up were assessed.

## PATIENTS AND METHODS

In the North Netherlands, the first guidelines for the treatment of patients with DCIS were developed in 1992 by a cooperating group of specialists in the field of breast cancer in the region (Otter, 1992). These clinical guidelines were based on evidence from existing literature and guidelines by other groups and have been updated every 2 or 3 years. According to the above-mentioned guidelines and to those of 1994 (Otter, 1994), BCS is advised in the case of a tumour smaller than or equal to 2 cm and a simple mastectomy in the case of a tumour larger than 2 cm. It is essential that BCS and mastectomy result in tumour-free margins. The 1996 guidelines (Otter, 1996) state that a complete surgical removal of all DCIS with microscopic free surgical margins is advised. Involvement of the deep margin after BCS is considered as a positive margin. A simple mastectomy is the safest way and BCS should consist of wide local excision with microscopic free margins. Adjuvant radiotherapy is optional but should not replace surgery for incomplete removal of DCIS. The 1998 (Otter, 1998) and 2000 (Otter, 2000) guidelines advise BCS with standard administration of radiotherapy for intermediately and poorly differentiated DCIS (Grades 2 and 3) and a simple mastectomy if no free margins can be achieved. If the excision is not complete in the case of well-differentiated DCIS, a wait-and-see policy is permitted if the patient is motivated, if there are no indications of hereditary breast cancer, and if mammograms can be properly reviewed. According to the 2003 guidelines (Otter, 2003), BCS and postoperative radiotherapy is advised for all subgroups. Chest wall radiation is advised if DCIS extends to the deep margin after a simple mastectomy. A sentinel node procedure could be performed in the case of tumours ⩾5 cm. Axillary lymph node dissection (ALND) has not been recommended in any guideline since 1992 and the administration of tamoxifen or other hormonal therapy has not yet been advised.

To investigate compliance with the guidelines and its effect on LR, 251 consecutive patients from two hospitals were studied. These patients had been treated for DCIS in the period January 1992 to December 2003 in the University of Groningen Medical Center (UMCG) and the Martini Hospital (MH). The UMCG is the only academic medical centre in the region and the MH is a large teaching hospital.

Data were retrieved retrospectively from medical charts and also from pathology and radiology reports. All analyses were performed on anonymous data. The present study is in agreement with the Dutch Law on the Conformity of Medical Treatment (WGBO). The following data were available for all patients: age, menopausal status, mode of detection, family history of breast cancer, mammographic appearance and size, fine-needle aspiration cytology, stereotactic large core needle biopsy or ultrasound guided large core needle biopsy (SCNB), treatment modalities, pathological size, pathological grade according to the European Pathologist Working Group classification (Shoker and Sloane, 1999), surgical margins and follow-up. Mammography and pathological characteristics were derived from radiology and pathology reports. If data were missing, mammography and pathological slides were re-evaluated. Surgical margins <1 mm were considered to be positive. LR was defined as ipsilateral breast or chest wall recurrence of both DCIS and invasive breast cancer. The date of last follow-up was November 2004. Compliance with guidelines was stated as follows: treatment was classified as appropriate (guidelines+) if the interventions undertaken were in agreement with the guidelines in operation at the time of treatment, whereas deviations from the guidelines were classified as inappropriate (guidelines−). Owing to changes in the guidelines, the total study period is divided into three separate time periods (1992–1995, 1996–1999 and 2000–2003).

Between January 1992 and December 2003, 1389 patients were treated for DCIS in the North Netherlands, an area of 2.1 million inhabitants. The main sources for cancer registration at the Comprehensive Cancer Center North Netherlands (CCCN) are the national computerised pathology databank (PALGA) and the hospital discharge databank to which all Dutch hospitals provide information annually on the discharge diagnoses of patients admitted. Specially trained CCCN employees prospectively register data regarding patients diagnosed with DCIS. Within the CCCN district, there is one academic medical centre, four teaching general hospitals and 12 nonteaching general hospitals.

As there are no data available in the CCCN cancer registry on tumour size and margin status, and because LR had only been registered if the recurrence consisted of invasive disease, it was not possible to evaluate all patients for treatment according to the guidelines. Therefore, clinical data concerning this population of patients treated for DCIS were used to determine whether the two-hospital sample was representative for the total region of the North Netherlands.

To evaluate the effect of compliance with existing guidelines, patients were divided into an LR positive (LR+) and an LR negative (LR−) group. Univariate Cox regression analyses were performed for each prognostic factor separately, considering the time to the onset of LRs as the outcome. Hazard ratios (HRs) and 95% confidence intervals (CIs) were estimated. To test the assumption of proportional hazards, an interaction term of a prognostic variable and a time-dependent covariate were added to each separate model (Klein and Moeschberger, 2003). A significant effect of that interaction term denotes the presence of a time-dependent effect and thus a violation of the proportional hazards assumption. As a control for unmeasured differences in the study period, due to changes in guidelines over time, we added the study period as a factor in the multivariate Cox regression analysis. Subsequently multivariate Cox regression analyses were performed. The elimination of variables in a stepwise manner identified the statistically significant predictors. LR-free survival was analysed using Kaplan–Meier survival analysis and the log-rank test. Patients were censored if they had died or otherwise been lost to follow-up. A *P*-value of 0.05 was considered as significant. All analyses were performed with program SPSS version 12.01.

## RESULTS

Clinicopathological characteristics of patients in the two-hospital sample are summarised in Table 1. The median age of the total study population was 57 years (range 32–85 years). In all, 49% (*n*=122) of all cases were detected by the Dutch Breast Cancer Screening Programme. Nearly one-third of the women (*n*=79) had not been treated according to the guidelines operating in the year of treatment. A total of 18 patients had positive margins after final surgery.

BCS (HR=10.328, 95% CI 2.907–36.693), positive margins (HR=4.790, 95%CI 1.696–13.531) and inappropriate treatment (guidelines− (HR=4.339, 95% CI 1.695–11.18)) were all characteristics associated with LR in univariate analysis (Table 1). There was no violation of the assumption of proportional hazards regarding the outcome and any prognostic factor (unpublished data). In Table 2, the independent predictors of LR after treatment of DCIS are shown: BCS without radiotherapy (HR=7.842, 95% CI 2.126–28.926) and deviation from the guidelines (HR=2.778, 95% CI 0.982–6.781). Although the guidelines changed with time, time was not a significant factor in the Cox analysis (HR=1.254, 95% CI 0.272–5.776 for study period 1992–1995 and HR=1.976, 95% CI 0.526–7.421 for study period 1996–1999).

**Figure 1:**
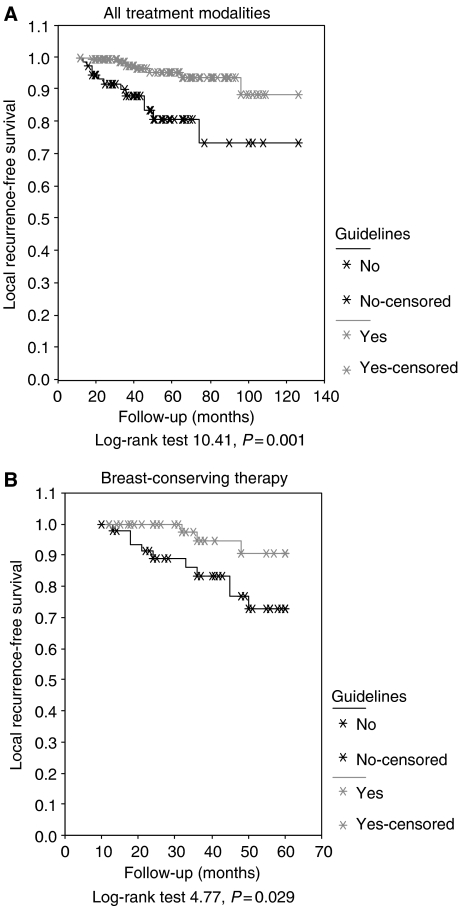
(**A**) Local recurrence-free survival in all patients who were treated according to the guidelines (guidelines+) and in all patients who were not treated according to the guidelines (guidelines−). (**B**) The 5-year local recurrence-free survival after BCS in patients who were treated according to the guidelines (guidelines+) and in patients who were not treated according to the guidelines (guidelines−).

The median follow-up was 43 months (mean 49, range 10–120 months). Figure 1A shows that LR-free survival was better in patients who had been treated according to the guidelines than in patients who had been treated inappropriately (log rank 10.41, *P*=0.001). The 5-year LR-free survival in patients treated with BCS, was 91% in patients who had been treated according to the guidelines and 73% in patients who had not been treated so (log rank 4.77, *P*=0.029; Figure 1B).

In Table 3, clinical data of patients treated in the two-hospital sample are compared with data from the entire population of patients in the North Netherlands. Patients in the two-hospital sample had undergone fewer axillary staging procedures than patients in the total region (*χ*^2^=32.64; *P*<0.001). Information on surgical management with regard to the entire population of patients in the North Netherlands was available for 1221 of the 1389 women involved: less than half of the women (*n*=525, 43.0%) had BCS. Information on axillary surgical management was also available for 1221 of the 1389 women involved: 299 women (24.5%) had ALND or SNB.

Of all patients with LRs (*n*=19) in the two-hospital sample, seven patients had been treated according to the guidelines and 12 patients had been treated inappropriately. Of these 12 patients, five had positive margins after final surgery, four had not received adjuvant radiotherapy after local excision, two patients had not undergone mastectomy, although their tumours were larger than two centimetres, and one patient had undergone ALND.

## DISCUSSION

Clinical guidelines reflect optimal management according to existing views and literature, and have been introduced to reduce inappropriate practice and to improve the quality of care (Lomas *et al*, 1989; Grimshaw and Russell, 1993; Mann, 1996). In the North Netherlands, the first guidelines for the treatment of patients with DCIS were developed in 1992 and subsequently they have been updated every 2 or 3 years. The present study was undertaken to investigate the effect of compliance with the existing guidelines on LR-free survival, and it clearly demonstrates that treatment according to these guidelines leads to a better outcome.

Owing to the change of guidelines over time, study period can be considered as a proxy for the nature of guidelines. However, since the study period is not a significant factor in the Cox analysis, the present study should not be considered to be a validation of the guidelines. In addition, the LR rate did not improve over time, since the events and follow-up of patients treated, especially in the third study period, are insufficient to show differences. Compliance with the guidelines is the only predictive factor, whereas there is no indicative effect of the nature of the guidelines on outcome. Other well-known risk factors for LR have been included in the study and they did not prove to be related with LR. Therefore, it can be concluded that there were no other factors in the group not treated according to the guidelines that could have led to their poorer outcome.

Physicians who carry out the treatment according to the guidelines often take a special interest in breast cancer, and they usually work in multidisciplinary teams. It is most likely that the positive effect of the guidelines on disease-free survival could be explained by this practice. It has already been demonstrated that surgeon workload is associated with survival from breast cancer (Stefoski Mikeljevic *et al*, 2003). If physicians are exposed to a high case volume of patients with DCIS, and if they follow the guidelines and work in multidisciplinary teams, it is plausible that they will be aware of the existing literature covering the whole process of diagnostic work-up, treatment and follow-up. This practice should result in better quality of care and an improvement in outcome.

Few data exist regarding outcome differences associated with deviation from clinical guidelines. Institutional validation of breast cancer treatment guidelines in Florida reported no effect from compliance with the guidelines on 5-year survival, but there was a significant reduction in the costs if the guidelines were followed (Minter *et al*, 2001). The above-mentioned study lacked statistical power to detect any small difference in overall 5-year survival between the groups. Furthermore, LR-free survival was not studied and patients with DCIS were excluded. In the systemic adjuvant treatment of patients with breast cancer, Olivotto *et al* (1994) demonstrated that improvements in disease-free survival and overall survival, noted during the time period of guideline implementation, were similar to those observed in clinical trials. The causality could not be demonstrated, but it was suggested that the improvements in disease-free survival were the result of operating within the guidelines.

Deviation from the guidelines automatically results in under- or overtreatment of patients. If patients have been undertreated, their prognosis is thought to be worse than that of patients who have been treated appropriately. Overtreatment leads to unnecessary procedures and overshooting the mark. The inappropriately treated population in the present study (*n*=79) consisted of 49 patients (62%) who had been undertreated, and 30 patients (38%) who had been overtreated. Under- or overtreatment was not associated with LR (unpublished data).

In the 12-year period 1992–2003, the guidelines of treatment of patients with DCIS have evolved, along with new evidence from studies and trials on this disease. If the most recent CCCN guidelines (Table 4) are compared with other guidelines (Rutgers *et al*, 2001, Olivotto *et al*, 2001; Morrow *et al*, 2002; The Association of Breast Surgery, 2005), conference reports (Schwartz *et al*, 2000; Senn *et al*, 2003) and review articles (Mokbel, 2003; Sakorafas and Farley, 2003; Burstein *et al*, 2004), there is a high degree of consensus between all these documents. Chest wall radiation for a positive margin after simple mastectomy is not mentioned in other guidelines. The administration of tamoxifen after BCS with radiotherapy (in oestrogen-receptor-positive patients) has so far not been issued, and this policy has not been followed in the two-hospital sample either. The addition of tamoxifen to the guidelines might help reduce the incidence of LRs.

The proportion of patients in the two-hospital sample who had been treated by simple mastectomy (52%) was similar to the proportion of patients in the entire region of the North Netherlands (57%). This percentage approximates the reports from earlier periods in the southeast Netherlands (1984–1989, 53%; Voogd *et al*, 2000) and from North Carolina (1990–2000, 52%; Kotwall *et al*, 2003), but is much higher than that reported in recent studies from California, Australia and Geneva (38, 24 and 22%, respectively; Morris *et al*, 2000; Verkooijen *et al*, 2002; Kricker and Armstrong, 2004). This indicates that mastectomy has played a dominant role in the treatment of patients with DCIS in the North Netherlands during the study period when compared with other studies. Axillary lymph node staging procedures (including sampling and SNB) had been performed on 299 (25%) out of 1221 patients (in 168 patients it was unknown whether axillary surgery had been performed). While this percentage was higher than in the subgroup of the two-hospital sample (11%), indicating that this percentage is rather high and could be brought down, it is comparable with data from parts of Australia (18%; Kricker and Armstrong, 2004) and the US SEER registries (15–34%; Baxter *et al*, 2004), indicating that, at the same time, it is acceptable to compare the percentages of our cohort of patients with DCIS to those of other large cohorts elsewhere.

Outcome in patients with DCIS was not measured as overall survival, but as LR-free survival because, in the follow-up period of the study, only two patients died. It was not possible to establish whether the two-hospital population under study was representative of the whole region with regard to pathological size and margin status after final surgery, or with regard to the number of LRs. This might yield bias in the sense that other factors associated with LR-free survival could have been identified had data of the entire population of patients of the North Netherlands been known.

In conclusion, the introduction and update of guidelines for the treatment of patients with DCIS in the CCCN region resulted in a compliance rate of 68.8% of the patients in the two-hospital sample and the two-hospital sample was representative for the whole region. Compliance with the guidelines was an independent predictor of disease-free survival. These data support the application of guidelines in the treatment of DCIS and they emphasise the importance of audit to assess whether guidelines are being followed.

## Figures and Tables

**Table 1:** Clinico-pathological characteristics of patients of the two-hospital sample and local recurrence (Cox's regression, univariate analysis)

**Clinicopathological characteristic**	**LR+ (*n*=19)**	**LR− (*n*=232)**	**HR**	**95% CI**	***P*-value**
Age (years) (mean)	60.4	57.3	—	—	—
⩽40 years	2 (10.5)	10 (4.3)	2.800	0.645–12.152	0.169
>40 years	17 (89.5)	222 (95.7)	1		
					
Premenopausal	3 (15.8)	60 (25.9)	0.411	0.118–1.436	0.164
Postmenopausal	16 (84.2)	172 (74.1)	1		
					
Screen detected	5 (26.3)	117 (50.4)	0.451	0.161–1.260	0.129
Other	14 (73.7)	115 (49.6)	1		
					
Family history	3 (15.8)	57 (24.6)	0.685	0.199–2.356	0.549
No family history	16 (84.2)	175 (75.4)	1		
					
FNAC	4 (21.1)	42 (18.1)	1.042	0.345–3.146	0.941
No FNAC	15 (78.9)	190 (81.9)	1		
					
SCNB	3 (15.8)	105 (45.3)	0.428	0.121–1.512	0.188
No SCNB	16 (84.2)	127 (54.7)	1		
					
Microcalcifications	8 (42.1)	61 (26.3)	1	0.661–4.126	0.283
No microcalcifications	11 (57.9)	171 (73.7)	1.651		
					
Density	12 (63.2)	165 (71.1)	1	0.375–2.450	0.929
No density	7 (36.8)	67 (28.9)	0.958		
					
Mammographic size ⩽2 cm	9 (47.4)	128 (55.2)	0.861	0.349–2.125	0.745
Mammographic size >2 cm	10 (52.6)	104 (44.8)	1		
					
BCS	13 (68.4)	50 (21.6)	10.328	2.907–36.693	<0.001
BCS+XRT	3 (15.8))	55 (23.7)	2.925	0.566–15.112	0.200
Mastectomy	3 (15.8)	127 (54.7)	1		
					
Axillary staging surgery	1 (5.3)	27 (11.6)	0.533	0.071–4.012	0.541
No axillary staging surgery	18 (94.7)	205 (88.4)	1		
					
Positive margins (<1 mm)	5 (26.3)	13 (5.6)	4.790	1.696–13.531	0.003
Negative margins (⩾1 mm)	14 (73.7)	219 (94.4)	1		
					
Pathological size ⩽2 cm	12 (63.2)	107 (46.1)	2.166	0.849–5.524	0.106
Pathological size >2 cm	7 (37.8)	125 (53.9)	1		
					
Grade 1	3 (15.8)	43 (18.5)	0.778	0.203–3.053	0.730
Grade 2	9 (47.4)	96 (41.4)	1.253	0.466–3.365	0.655
Grade 3	7 (36.8)	93 (40.1)	1		
					
Guidelines−	12 (73.2)	67 (28.9)	4.339	1.695–11.108	0.002
Guidelines+	7 (36.8)	165 (71.1)	1		
					
1992–1995	5 (26.3)	47 (20.2)	1.254	0.272–5.776	0.772
1996–1999	11 (57.9)	83 (35.8)	1.976	0.526–7.421	0.313
2000–2003	3 (15.8)	102 (44.0)	1		

LR+=local recurrence; age is depicted as the median value; HR=hazard ratio; CI=confidence interval; FNAC=fine-needle aspiration cytology; SCNB=stereotactic large core needle biopsy; BCS=breast-conserving surgery; BCS+XRT=breast-conserving surgery and radiotherapy; pathological grade according to EPWG classification; guidelines+=treatment according to CCCN guidelines.

**Table 2:** Predictors of local recurrence (Cox's regression, multivariate analysis)

**Predictors of local recurrence**	**HR**	**95% CI**	***P*-value**
BCS	7.842	2.126–28.926	0.002
BCS+XRT	2.432	0.471–12.552	0.085
Mastectomy	1		
Deviation from the guidelines	2.778	0.982–6.781	0.041
Compliance with the guidelines	1		

BCS=breast-conserving surgery; HR=hazard ratio; CI=confidence interval; BCS+XRT=breast-conserving surgery and radiotherapy.

Regression analysis by elimination of variables in a stepwise manner. As a control for unmeasured differences in the study period, due to changes in guidelines over time, the study period was added as a factor in the analysis.

**Table 3:** Clinical characteristics of patients of the entire region of the North Netherlands (CCCN population) and the two-hospital sample

**Clinical characteristics**	**CCCN population (*n*=1389)**	**Two-hospital sample (*n*=251)**	***χ* ^2^**	***P*-value**
Mean age (years) (range)	60 (22–98)	57 (32–85)	—	—

*Screen detected*				
Yes	625 (45.0)	122 (48.6)	1.12	0.291
No	764 (55.0)	129 (51.4)		
				
*Surgery*				
BCS	525 (43.0)	121 (48.2)	2.30	0.130
Mastectomy	696 (57.0)	130 (51.8)		
Unknown	168	0		
				
*Axillary surgery*				
ALND	240 (19.7)	12 (4.8)	32.64	<0.001
SNB	59 (4.8)	16 (6.4)		
No axillary	922 (75.5)	223 (88.8)		
				
*Surgery*				
Unknown	168	0		
				
*Radiotherapy*				
Yes	292 (55.6)	58 (48.0)	2.34	0.126
No	233 (44.4)	63 (52.0)		

BCS=breast-conserving surgery; ALND=axillary lymph node dissection; SNB=sentinel node biopsy.

Numbers between parentheses are percentages. Age is depicted as median value.

**Table 4:** Summary of recent clinical guidelines and recommendations in the management of DCIS

**Guidelines**	**BCS**	**Mastectomy**	**Axillary staging**	**Radiotherapy**	**Hormonal therapy**
EUSOMA (Rutgers *et al*, 2001)	BCS is advised in the case of small areas of DCIS (<3 cm) Condition: free margins	Mastectomy is advised in the case of large areas of DCIS (>3 cm) Mastectomy is advised in the case of persistent positive margins after BCS	Axillary staging is not recommended	Whole-breast irradiation is optional after BCS	—
					
SCCPG (Olivotto *et al*, 2001)	BCS is advised in the case of small areas of DCIS Conditions: cosmetically acceptable and free margins	Mastectomy is advised in the case of large or diffuse areas of DCIS Mastectomy is advised in the case of persistent positive margins after BCS	Axillary staging is not recommended	Whole-breast irradiation is advised after BCS	Hormonal therapy is optional after BCS and XRT
					
ACR ACS CAP SSO (Morrow *et al*, 2002)	BCS is advised in the case of localised DCIS and extent ⩽4 cm Conditions: cosmetically acceptable and free margins	Mastectomy is advised in the case of multifocal and diffuse DCIS Mastectomy is advised in the case of persistent positive margins after BCS	SNB or level I ALND is advised in the case of large areas of DCIS requiring mastectomy	Whole-breast irradiation is advised after BCS	Hormonal therapy is optional after BCS and XRT
					
CCCN (Otter, 2003)	BCS has the preference overmastectomy Conditions: cosmetically acceptable and free margins	Mastectomy is advised in the case of persistent positive margins after BCS	SNB is advised in the case of large areas of DCIS (⩾5 cm)	Whole-breast irradiation is advised after BCS Chest wall irradiation is advised in the case of a positive margin after mastectomy	Hormonal therapy is not recommended
					
BASO (The Association of Breast Surgery, 2005)	—	Mastectomy is advised in the case of extensive microcalcifications on mammography	SNB is advised in the case of extensive tumour, high grade, palpable mass or mass on mammography	Whole-breast irradiation is advised after BCS	—

BCS=breast-conserving surgery; EUSOMA=European Society of Mastology; condition=all the conditions must be met in order to carry out BCS; SCCPG=Steering Committee on Clinical Practice Guidelines for the Care and Treatment of Breast cancer; ACR=American College of Radiology; SNB=sentinel node biopsy; ACS=American College of Surgeons; ALND=axillary lymph node dissection; CAP=College of American Pathology; SSO=Society of Oncology; CCCN=Comprehensive Cancer Centre North Netherlands; BASO=British Association of Surgical Oncology.
